# Current understanding of the role of DNA methylation in pituitary neuroendocrine tumors

**DOI:** 10.1093/noajnl/vdae149

**Published:** 2025-01-02

**Authors:** Racheal Peterson, David J Cote, Gabriel Zada

**Affiliations:** Department of Neurosurgery, Keck School of Medicine, University of Southern California, Los Angeles, California, USA; Department of Neurosurgery, Keck School of Medicine, University of Southern California, Los Angeles, California, USA; Department of Neurosurgery, Keck School of Medicine, University of Southern California, Los Angeles, California, USA

**Keywords:** epigenetics, methylation, pituitary neuroendocrine tumor, pituitary adenoma

## Abstract

Pituitary neuroendocrine tumors (PitNETs) are unusual among neoplasms in that sporadic tumors are not typically associated with genetic mutations. Instead, epigenetics, a non-mutational process by which gene expression is modified via a variety of mechanisms, may be a driving factor in PitNET growth and behavior. DNA methylation is one of the most well-understood forms of epigenetic modification. Research on DNA methylation profiles of PitNETs has identified a large number of genes silenced or upregulated by DNA methylation, particularly when methylated at CpG islands of gene promoter regions. Global patterns of DNA methylation may provide valuable insight into the origins of pituitary adenoma subtypes, assist with PitNet diagnostics, and have been found to correspond to the current World Health Organization classification of PitNETs based on transcription factor lineage. Analysis of differentially methylated regions of individual genes may have prognostic value as well as guide research toward nonsurgical therapeutic strategies. Pituitary epigenetics and DNA methylation analysis are rapidly growing areas of interest with the potential to shape the future of pituitary tumor diagnostics and treatment.

## Background

Pituitary neuroendocrine tumors (PitNETs, formerly labeled pituitary adenomas [PAs]) are predominantly indolent tumors representing 10%–15% of intracranial neoplasms,^1,2^ and may occur incidentally in as high as 20% of the population.^3–5^ They arise from a line of hormone-associated precursor cells and are currently classified by the World Health Organization (WHO) according to their transcription factor-associated lineage (PIT-1, SF-1, and T-PIT).^4–6^ Up to 35% of PAs are hormonally inactive “nonfunctional” PitNETs; however, clinically functional PitNETs secrete hormones reflective of their lineage.^5^ Most nonfunctional PitNETs arise from the gonadotroph SF-1 transcription factor lineage, although “silent” subtypes of PIT-1 and T-PIT PAs also arise not infrequently. Nonfunctioning gonadotroph tumors make up the majority of nonfunctioning PitNETs.^6^ Corticotroph tumors, including the aggressive Crooke cell subtype, are classically small-sized tumors of the T-PIT lineage that usually secrete excess adrenocorticotrophic hormone (ACTH), leading to hypercortisolemia and Cushing’s disease.^7^ Corticotroph PitNETs are the second most common type of clinically silent PitNET; however, for the purpose of this review will be referred to as silent corticotroph PitNETs to distinguish from nonfunctioning gonadotroph PitNETs (NF-PitNETs).^2^ Somatotroph tumors are tumors derived from somatotropic cell lines that secrete excess growth hormone (GH), stimulating an increase in insulin-like growth factor 1 (IGF-1) and leading to acromegaly and abnormal bone, tissue, and organ growth.^7^ Lactotroph tumors, or prolactinomas, makeup 60% of all PitNETs and usually exhibit slow, stable growth patterns.^8^ Rare thyrotroph tumors comprise only 1% of all PitNETs and can lead to elevated or suppressed thyrotropin levels with normal or elevated thyroid hormone levels.^7,8^ Somatotroph, lactotroph, and thyrotroph tumors derive from the PIT-1 lineage.^6^ Plurihormonal tumors exhibit any combination of the above transcription factors, while null cell tumors are those with no transcription factor identified.^2^

In addition to hormonal dysfunction, PitNETs can also cause morbidity due to mass effect (optic chiasm compression leading to visual disturbance; hypopituitarism due to compression of the normal pituitary gland) or local invasion (cranial nerve dysfunction due to cavernous sinus involvement), although the majority are <10 mm in diameter and limited to the sella.^5^ Less than 1% of all pituitary tumors result in metastasis.^1,3^

Aggressive pituitary tumors are characterized by local invasion, rapid rate of growth, and/or continued growth despite standard treatment with surgery, radiation, and medical treatment.^9^ Identification of patients harboring aggressive as compared to more clinically indolent PitNETs remains an area of active research interest, given the importance such a distinction could play in the future management of these tumors.

Although rare, certain familial PitNETs are associated with mutations in the multiple endocrine neoplasia I (MEN1) or aryl hydrocarbon receptor-interacting protein (AIP) genes.^5,10^ Sporadic pituitary tumorigenesis, however, in contrast to other types of neoplasms, has not been strongly linked to genetic mutations; the few mutations described include AIP, guanine nucleotide-binding protein, alpha stimulating (GNAS), and ubiquitin-specific protease 8 and 48 (USP8/48).^1,11–13^ These genetic mutations and hereditary disorders associated with PitNET growth have been described elsewhere in the literature, allowing this review to focus on the epigenetic changes seen in pituitary tumors. Epigenetic alterations have been suggested to play a role in the regulation of tumor growth, behavior, and treatment response, and as such are a growing area of research interest.^5,14,15^

### Pituitary Tumor Genetics and Epigenetics

Epigenetics is a diverse group of processes that influence gene expression without modifying the underlying DNA sequence.^16^ Mechanisms by which gene expression can be modified include DNA methylation, histone modification, gene imprinting, epigenome writers, and transcription regulators.^1^ In the normally developing pituitary gland, epigenetic modifications control the process by which cells sharing a common progenitor and genome can exhibit distinct cell types and functions.^17^ While these processes are a component of normal cellular function, dysregulation can lead to the uncontrolled cell growth seen in tumorigenesis.^1,18^ Gene-associated CpG island methylation, particularly at gene promoter regions, plays a role in gene silencing, while histone tail modifications such as methylation, acetylation or phosphorylation can lead to silencing or upregulation of gene expression.^16,18^ Recent studies in pituitary epigenetics have shown several alterations in DNA methylation levels that contribute to tumor aggressiveness and response to treatment.^5,15^ The purpose of this review is to describe the current understanding of pituitary epigenetics subject to DNA methylation, and the clinical impact of DNA methylation in the management of PitNETs.

## DNA Methylation

DNA methylation is a reversible epigenetic process that plays a role in many tumorigenic states, typically through transcriptional silencing.^14,16^ The process is primarily catalyzed by DNA methyltransferase (DNMT) enzymes at CpG dinucleotides, also known as CpG islands.^19^ Hypermethylation of CpG islands classically leads to reduced expression of known tumor suppressor genes in PitNETs.^5^ The first genome-wide analysis of the pituitary genome and epigenome was published in 2012 and studied methylation status at over 27 000 CpG sites in nearly 14 000 genes.^18^ A subsequent genome-wide study of DNA methylation profiling identified 34 CpG sites on 17 genes showing significant variability in methylation levels between nonfunctional and functional PitNETs.^5^ Later reports have corroborated the varying DNA methylation patterns seen in different PitNET subtypes; however, DNA methylation alone is not always associated with reduced expression and may be influenced or reinforced by histone tail modifications or other contextual modulation.^5,14,16^ Histone deacetylases (HDACs) are recruited through the methyl-DNA binding motifs of several HDAC complex components. Histone methylation may be a requirement for DNA methylation, or at a minimum have a reinforcing relationship.^14^

While genome-wide profiling has provided insight into the global methylation patterns of PitNETs, many studies focus on specific genes with oncological ramifications in hopes of providing insight into tumor behaviors or guiding future drug development. Epigenetic changes can vary by tumor subtype and are more frequent in NF-PitNETs.^18^ Altered DNA methylation status has been seen in cell cycle regulators, signal transduction pathways, apoptosis regulators, developmental genes, and growth factor signaling components, leading to dysregulated cell growth and signaling by a non-mutational mechanism.^18,19^

Frequencies of DNA methylation can vary widely among genes, with many still requiring further investigation to characterize fully. Genes demonstrating frequent DNA methylation (present in over 50% of PitNETs) include *CDKN2A, GADD45γ*, *FGFR2, CASP8,* and *Pituitary tumor apoptosis gene (PTAG)*.^1,20^ Moderate DNA methylation levels (20%–50% of PAs) are seen with *TSP-1, RASSF1A, RB1, p73, MGMT,* and *CDH1.*^1,20^ Other genes such as *CDKN2A, CDKN1A, CDKN1B, Death associated protein kinase 1 (DAPK1), and TIMP3* are methylated in less than 20% of PitNETs.^1,20^ An overview of genes subject to DNA methylation is shown in Table 1.

**Table 1.** Non-comprehensive List of Genes Subject to DNA Methylation in Pituitary Adenomas With Relative Methylation Frequencies and Commonly Associated PA Subtypes. Frequent: >50% Methylation; Moderate: 20%–50% Methylation; Low: <20% Methylation

Gene	Normal function	Methylation frequency in PAs	Associated PA subtypes
GADD45γ	Cell cycle regulation	Frequent	All subtypes
PTAG	Apoptosis	Frequent	All subtypes
CDKN2A/p16	Cell cycle regulation	Frequent	Nonfunctional
MEG3	Tumor suppressor	Frequent	Nonfunctional
CASP8	Apoptosis	Frequent	Corticotroph, somatotroph
BMP-4	Cell signaling	Frequent	Somatotroph, lactotroph, and corticotroph
p15	tumor suppressor	Moderate	All subtypes
p73	Cell cycle regulation and apoptosis	Moderate	All subtypes
EFEMP1	Cell signaling	Moderate	All subtypes
MGMT	DNA Repair	Moderate	All subtypes
FGFR2	Growth factor signaling	Moderate	All subtypes
TSP-1	Cell signaling	Moderate	All subtypes
RB1	Cell cycle regulation	Moderate	Somatotroph
RASSF1A	Tumor suppressor	Moderate	Somatotroph, lactotroph, and corticotroph
CDH1	Cell signaling	Moderate	Invasive, all subtypes
CDH13	Cell signaling	Moderate	Invasive, all subtypes
p21	Cell cycle regulation	Low	All subtypes
p27	Cell cycle regulation	Low	All subtypes
LGALS3	Cell growth and differentiation	Low	All subtypes
TIMP3	Apoptosis	Low	Nonfunctional
POMC	Hormone function	Low	Corticotroph
ESR1	Hormone function	Low	Corticotroph
GNAS	Hormone function	Low	Somatotroph
AIP	Apoptosis	Low	Invasive, all subtypes
DAPK1	Apoptosis	Low	Invasive, all subtypes
PDCD1	Apoptosis	Variable	Low in aggressive subtypes

DNA methylation signatures in pituitary tumors are characteristic of their lineage, with distinct methylation profile clusters that associate with transcription factor lineage and the current WHO classification recommendation.^2,21–23^ Globally, PIT1-derived tumors demonstrate low levels of DNA methylation, SF1-derived PitNETs are hypermethylated, and TPIT-derived tumors are intermediately methylated relative to the other subtypes. Methylome-wide analysis comparing PitNETs has also allowed for the identification of DNA methylation signatures at distinct CpG sites (mostly CpG islands at gene promoter regions), known as differentially methylated probes (DMPs), that are exclusive to selected PitNET subtypes.^23^ Significant efforts are underway to develop a database of DNA methylation profile information for all central nervous system tumors including PitNETs in order to improve the classification of tumors beyond WHO categories by standardizing diagnostic criteria and reducing inter-observer variability.^24^ In the future, DNA methylation (and possibly blood-borne diagnostics) of cell-free DNA methylation markers may play an increasing role in diagnostics and prognostics for PitNETs.^25^

### Cell Cycle Regulation and Signal Transduction

*CDKN2A* is a tumor suppressor gene that encodes p16 and has been associated with deletions, point mutations, and methylation inhibition in many tumor types. In PitNETs, *CDKN2A* methylation is seen in the majority of nonfunctional gonadotroph PitNETs compared to a few somatotroph or corticotroph tumors, and it is not methylated in normal pituitary tissue.^26^ Methylation of *CDKN2A* triggers a cascade in which decreased p16 expression leads to phosphorylation of retinoblastoma protein, which activates E2F transcription factors to enable cell cycle progression.^17^*CDKN2C* has also been associated with promoter methylation, which decreases expression of p18 [27].

In addition to decreased expression via phosphorylation from the p16/CDKN2A cascade, retinoblastoma tumor suppressor gene (*RB1*) silencing via hypermethylation is present in 27% of PitNETs, and it is more frequent in somatotroph tumors than NF-PitNETs.^20,27–29^ The retinoblastoma pathway of cell cycle regulation itself is affected by the expression of several genes including *RB1*, *p15*, and *p16/CDKN2A*, and studies have shown that at least 90% of PitNETs demonstrate methylation-induced silencing of at least one RB-pathway gene.^20,29^ This suggests that epigenetic modulation of the RB pathway plays a significant role in pituitary tumorigenesis, but other pathways also contribute to human PitNET growth.^29^

*P15* is a tumor suppressor gene commonly found to have mutations in a wide variety of human tumors; however, these mutations have not been identified in pituitary tumors.^20^*P14*, despite proximity and similar functioning to *p15* and *p16/CDKN2A*, displays very low levels of methylation in PitNETs.^20^*P21* and *p27* are cyclin-dependent kinase inhibitors that prevent cell cycle progression to suppress tumor growth.^20^ Loss of *p27* is associated with pituitary tumor growth and progression.^14^ DNA methylation of *p21* and *p27* has been seen very rarely, suggesting that this is not the main mechanism for the loss of function of these genes, which may rather be susceptible to post-translational ubiquitin-mediated degradation.^14,20^*P73* encodes a protein similar to p53 that induces apoptosis and cell cycle arrest via activation of *p21* transcription.^20^ Hypermethylation and loss of function have been observed in a small but significant fraction of PitNETs compared to normal pituitary tissue.^20^

Growth arrest and DNA-damage inducible gene (*GADD45γ*) negatively regulates cell growth and is involved in DNA damage repair.^7^ Methylation of *GADD45γ* is associated with reduced transcription leading to cell growth and tumorigenesis, though it is not the sole mechanism responsible for *GADD45γ* silencing.^14,30,31^*GADD45γ* expression is suppressed in most PitNETs, however only about half of these demonstrate methylation-mediated silencing.^30,31^

Both E-cadherin (*CDH1*) and H-cadherin (*CDH13*) encode proteins that play a critical role in cell–cell adhesion.^32^ Downregulation via promoter hypermethylation is tumor-specific and found in all tumor subtypes.^32^ Methylation is associated with invasive behavior in PitNETs and may play a role in predicting tumor aggressiveness.^32^

The Ras-association domain family 1 (*RASSF1A*) gene is a tumor suppressor with hypermethylation seen frequently in malignant breast, lung, genitourinary, gastrointestinal, and sinonasal cancers.^33^ Promoter region methylation of *RASSF1A* has variable frequency among PITNET subtypes and is most common in somatotroph, lactotroph, and corticotroph tumors.^33^ It can be detected in both aggressive and non-aggressive tumors; however, there is a slight correlation between Ki-67 and methylation status.^7,33^

EGF-containing fibulin-like extracellular matrix protein 1 (*EFEMP1*) gene encodes fibulin-3, an extracellular matrix protein with both tumor promoter and tumor suppressor properties. *EFEMP1* is hypermethylated in most tumors regardless of subtype, leading to decreased *EFEMP1* expression. This decreased expression is due to a combination of both CpG methylation and histone modification.^16,18^

### DNA Repair

O^6^-methylguanine DNA methyltransferase (*MGMT*) methylation is well known to affect glioblastoma behavior and response to therapy and may have implications in PitNETs, as well.^34^ Approximately 40% of both functional and nonfunctional PitNETs exhibit *MGMT* methylation, which is associated with a lower rate of tumor regrowth.^1,34^

### Apoptosis

Death associated protein kinase 1 (*DAPK1*) is a positive mediator of apoptosis that normally functions to restrict tumor growth.^35^ Loss of *DAPK1* function via methylation is seen in a low percentage of PitNETs overall but is significantly associated with invasive tumors.^35,36^ Pituitary tumor apoptosis gene (*PTAG*) induces apoptosis through caspase-mediated pathways.^37^ Methylation of *PTAG* increases cell resistance to apoptosis and is a frequent finding in pituitary tumors.^37,38^ Caspase 8 (*CASP8*) also plays a role in apoptosis and is often hypermethylated in corticotroph-secreting tumors.^28^

The tissue inhibition of metalloproteinase 3 (*TIMP3*) gene suppresses tumor growth by antagonizing extracellular matrix metalloproteinases.^39^*TIMP3* methylation is relatively low in PitNETs with a trend toward higher methylation levels in NF-PitNETs than functional PitNETs.^28^

The aryl hydrocarbon receptor-interacting protein (*AIP*) gene is a tumor suppressor gene that has been associated with a variety of mutations in certain familial pituitary tumors.^29^*AIP* likely suppresses tumor growth via activation of the aryl hydrocarbon receptor (AHP), which inhibits proliferation or induces apoptosis through several downstream mechanisms, including activation of the *RB* pathway.^29^ Non-mutational silencing of *AIP* via methylation is most commonly associated with somatotroph tumors but can be seen in all PitNET subtypes.^7^ Differences in methylation level based on tumor behavior have been observed, with aggressive or invasive PitNETs showing lower levels of AIP methylation.^15^

Maternally expressed 3 (*MEG3*) is an imprinted long noncoding RNA (lncRNA) tumor suppressor that regulates the expression of the p53 gene (*TP53*), another tumor suppressor gene.^1,40^ Methylation of *MEG3* in NF-PitNETs leads to silencing of *MEG3* gene expression and loss of its regulation on p53.^1^

The programmed cell death protein 1 (*PDCD1*) gene encodes an immune-inhibitory receptor expressed by activated T cells that engages with programmed death ligand 1 (PD-L1) on tumor cells to allow cancers to escape a normal T-cell mediated apoptosis checkpoint and proliferate.^41^ Higher levels of PD-L1 protein and mRNA have been seen in functional and aggressive PitNETs compared to NF-PitNETs and non-aggressive tumors.^42^*PDCD1* methylation levels are lower in aggressive PitNETs, making these tumors more capable of bypassing the immune checkpoint.^15^

### Development and Growth Factor Signaling

Fibroblast growth factor (FGF) is a family of 23 ligands whose signaling is crucial to pituitary development, and several FGF ligands are overexpressed in pituitary tumors. FGF2 is involved in stem cell maintenance and neurogenesis during embryonic development and during periods of stress or injury in the adult brain and is crucial for normal pituitary development.^14,17^ Downregulation of the FGF2 receptor (*FGFR2*) gene via promoter methylation leads to reduced growth factor receptor expression, allowing tumor cells to progress through the cell cycle.^7,14^ Pituitary tumor tissue contains downregulated *FGFR2* in 52% of PitNETs, with methylation found in 45%.^14^

Bone morphogenic protein (*BMP*)-4 is a multifunctional secretory peptide that has autocrine and paracrine roles in anterior pituitary development.^43^ The *BMP-*4 gene is upregulated in prolactinomas and decreased in other tumor subtypes compared to normal pituitary tissue.^43^*BMP-4* has a cell-type specific context and has been associated with the stimulation of tumor growth in lactotroph and somatotroph tumors and the inhibition of corticotroph growth.^43^ Despite the differing clinical effects of *BMP-4*, corticotroph, and somatotroph cell lines both show heavy *BMP-4* methylation and reduced expression compared to normal pituitary tissue.^43^

Thrombospondin-1 (*TSP-1*) encodes an adhesive glycoprotein that is involved with platelet aggregation, angiogenesis, and tumorigenesis via the mediation of cell–cell and cell-matrix interactions.^7^*TSP-1* also plays a role in antagonizing *BMP- 4*.^44^ Moderate levels of *TSP-1* methylation are detected across PitNET subtypes.^5^

Galectin-3 is a protein encoded by *LGALS3* that is involved in a variety of cellular functions including growth, differentiation, adhesion, angiogenesis, tumor progression, apoptosis, and metastasis, and is highly expressed in many human cancers.^45^ Methylation of *LGALS3* decreases galectin-3 expression in PitNETs; however, not all PitNETs with low galectin-3 expression are methylated.^45^ Galectin-3 expression has been associated with aggressive behavior in PitNETs.^45^

## Clinical Relevance

### Differential Methylation Based on Pituitary Tumor Subtype

DNA methylation patterns and transcription factors in PitNETs play a role in the terminal differentiation of tumor subtypes. Recent studies in pituitary transcriptomics have demonstrated 3 distinct subtypes of PitNET clusters that also correspond to global methylation patterns.^13,46^ Pituitary-specific positive transcription factor 1 (PIT-1, also known as POU1F) drives the growth of GH-, PRL-, and TSH-secreting tumors.^6,10^ These functional PitNETs share common methylation patterns, which can be further subdivided based on the affected hormone.^6^ T-box transcription factor 19 (T-PIT, or TBX19) is associated with ACTH tumors, which have about 649 genes affected by DNA methylation.^6,10^ NF-PitNETs, which include most gonadotroph tumors, are associated with steroidogenic factor 1 (SF-1, or NR5A1) with approximately 1066 genes subject to DNA methylation.^6,10^ The overall hypermethylated state of nonfunctioning (including gonadotroph and corticotroph) PitNETs has been hypothesized to be one mechanism by which hormone production is suppressed in these tumor types, due to the general effect of genetic silencing caused by DNA methylation (mostly at gene promoter sites of tumor suppressor genes).^23,47^ GH-secreting tumors tend to be overall hypomethylated compared to ACTH-secreting or hormonally inactive tumors.^13^ Silent corticotroph tumors, which demonstrate positive immunostaining for ACTH without clinical Cushing’s disease, demonstrate methylation characteristics of both ACTH-PitNETs and gonadotroph-derived NF-PitNETs.^10^

Several genes demonstrate variable DNA methylation levels in corticotroph tumors, which encode a variety of functions, including hormone synthesis, apoptosis, and cell cycle progression. It is possible that epigenetics plays a role in differentiating silent from functional corticotroph tumors. Our current understanding of epigenetic modulation in corticotroph tumors stems from studies on human-derived tumor tissue as well as mouse-derived AtT20 cell lines.

The proopiomelanocortin (*POMC*) gene encodes the precursor of ACTH and is often hypomethylated in corticotropin-secreting tumors, leading to *POMC* upregulation and increased secretion of ACTH.^7^ Increased expression of TSP-1 also leads to increased secretion of ACTH.^48^*CASP8* demonstrates higher levels of DNA methylation in corticotroph-secreting tumors compared to silent corticotroph tumors, suggesting a functional effect of *CASP8* methylation, but the mechanism by which this modulation occurs is not well understood.^7,21^*ESR1* encodes an estrogen receptor and is co-expressed with *POMC* in the hypothalamus, possibly playing a role in hypothalamic *POMC* expression.^21^ Methylation of *ESR1* is higher in functional corticotroph tumors compared to silent corticotroph tumors, but it is currently unknown whether this relates to hormonal function.^7,21^

The guanine nucleotide-binding protein alpha stimulating (*GNAS*) gene encodes the alpha-subunit of GTP-binding protein.^49^ Mutations in *GNAS* result in the production of the *gsp*-oncogene, which has been demonstrated to occur in somatotroph tumors.^49^*GNAS* can also be subject to epigenetic changes such as loss of methylation, which can decrease *GNAS* expression, leading to decreased expression of somatostatin receptor 2 (*SSTR2*) and *AIP*.^49^*GNAS* may play a role in somatotroph tumor sensitivity to somatostatin inhibitor therapy.

Null cell tumors are rare pituitary tumors that were previously identified as those lacking hormonal immunostaining; however, with the use of transcription factor staining, up to 80% of these tumors may demonstrate a clear lineage, increasing the rarity of true null cell tumor incidence.^2,50^ To date, true null cell PitNETs have not shown a distinct methylation pattern, although this is likely due to their lack of representation in larger studies.^50^ In contrast to null cell PitNETs, transcription factor staining may reveal an increase in the number of PitNETs of multiple lineages, or plurihormonal PitNETs.^51^ One recently published study examining the molecular profiles of SF-1/PIT-1 plurihormonal PitNETs revealed distinct profiles compared to each individual subtype; however, no studies to date have examined the epigenetic characteristics of plurihormonal tumors.^51^ Similarly, while Crooke’s cell PitNETs may have low levels of MGMT expression, there are currently no published studies on their methylation profiles and how they may differ from other functional or silent corticotroph PitNETs.^52,53^

### DNA Methylation Associations With Tumor Behavior

DNA methylation may also provide insight into tumor behavior. Variable methylation levels leading to altered oncogene and onco-suppressor expression may be an early event in determining tumor aggressiveness, metastasis, and response to treatment.^15^ Lower levels of DNA methylation have been shown in *AIP*, *GNAS*, and *PDCD1* in aggressive compared to non-aggressive PitNETs.^15^ Aggressive PitNETs and metastatic PitNETs have been associated with higher levels of DNA methylation of *PARP15*, *LINC0059*, and *ZAP70* compared to non-aggressive PitNETs.^15^ Inhibitor of growth family member 2 (*ING2*) is a growth inhibitor involved in DNA repair and apoptosis via regulation of histone deacetylase and histone acetyltransferase complexes.^7^*ING2* methylation levels may serve as a prognostic marker for NF-PitNETs, and high levels have been found to independently predict tumor regrowth.^54^ Family with sequence similarity 90 member A1 (*FAM90A1*) methylation levels have similarly been correlated with prognosis, and low levels also independently predict regrowth.^54^ Methylation levels of *FAM90A1* and *ING2* demonstrate specificity for NF-PitNETs and may become useful as prognostic markers.^7,54^

Several x-linked genes have also demonstrated DNA methylation, which may contribute to variable behavior in male and female patients. In males, higher methylation levels have been seen in *FLNA*, *UXT*, and *MAGE* family genes in association with aggressive PitNETs.^15^ For the same genes, tumor aggressiveness was not associated with stratified methylation levels in female patients, possibly due to genetic redundancy.^15^

Emerging data shows that high methylation levels of *PARP15*, *LINC0059*, and *ZAP70* or low methylation of *AIP*, *GNAS*, and *PDCD1* are associated with aggressive tumor behavior.^15^ Some x-linked genes such as the *MAGE* family, *UXT*, and *FLNA*, which play a role in regulating androgen receptors, demonstrate differential methylation patterns according to patient gender, with hypermethylation present in males but not females, and may account for differing tumor behaviors in male and female patients.^15,55^

### Therapeutic Targets

The reversible nature of epigenetic changes has made them the focus of new so-called “epidrugs” which target DNA methylation or histone tail modifications and restore the function of the silenced genes.^16^ Currently investigated epidrugs have utility both as standalone agents and in augmenting conventional therapies.^16^ Combining methylation analysis with drug repositioning studies may help identify new uses for known drugs.^56^

Zebularine (a DNA demethylating agent) and trichostatin A (TSA, a histone deacetylase inhibitor) induce robust re-expression of *BMP-4* when used in combination, compared to a modest increase for each individually, and actually leads to decreased methylation of *BMP-4* [43]. This combination also induces dose-dependent re-expression of *EFEMP1* with a marginal decrease in methylation, suggesting the histone modification effects of TSA may play an important role in this interaction.^16,57^

Aggressive PitNETs demonstrate low levels of *PDCD1* methylation and high levels of PD-L1 protein expression.^42^ This increased expression suggests there may be a role for PD-L1 inhibitors such as nivolumab or pembrolizumab.^15,42^ Anti-*PDCD1* drugs may also be effective for PitNETs with *PDCD1* methylation, but the lower methylation levels in aggressive PitNETs bypass this potential intervention.^15^

Temozolomide is an alkylating chemotherapeutic agent that methylates DNA to counteract the effects of *MGMT* and is currently used in the treatment of malignant gliomas.^58^ Studies have suggested that there may be a role for temozolomide (TMZ) therapy in aggressive PitNETs with unmethylated *MGMT* that may be prone to recurrence.^34^ High levels of *MGMT* methylation correlate with poor response to TMZ in metastatic PitNETs, similar to what is observed in gliomas^58,59^ However, *MGMT* staining does not correlate with temozolamide response as closely in PitNETs. Several case reports have had mixed success with using temozolamide to achieve ACTH reduction and long-term remission of Cushing’s disease in patients with MGMT-negative Crooke’s cell PitNETs.^52,53,60,61^ The use of TMZ in the treatment of PitNETs appears to be less straightforward than its use in gliomas and requires further research to determine when it may be appropriate.

Retinoic acid has previously been shown to inhibit tumor cell proliferation in a mouse model of Cushing’s disease, a finding that was accompanied by decreased levels of ACTH and cortisol.^62^ Retinoic acid pharmacologically inhibits cell proliferation and ACTH secretion in the AtT-20 pituitary corticotroph cell line via induction of *BMP-4* expression,^63^ however this mechanism still appears to be mediated by epidrugs and augmented by retinoic acid.^16^

Epidrugs may also serve to augment response to other therapies. Studies have suggested that 5-aza-deoxycytidine (decitabine), a DNA demethylating agent, increases expression of the somatostatin receptor 2 (*SSTR2*), leading to increased uptake of the somatostatin analog octreotide.^64^ Forced expression of *PTAG* leads to increased sensitivity to bromocriptine-induced apoptosis and may improve prolactinoma response to medical therapy.^38^

### Future Applications

Our understanding of pituitary epigenetics and the role of DNA methylation has expanded significantly over the last 2 decades, yet much remains to be discovered. Analysis of global methylation patterns may provide better insight into tumor behavior and help predict which tumors are likely to exhibit recurrence or aggressive growth. A DNA methylation analysis tool developed by the German Cancer Research Center (DKFZ) allows researchers to upload methylation profiles analyzed using Illumina Infinium HumanMethylation450 BeadChip (450K) array to compare to a database of over 2800 CNS tumor methylation profiles.^24^ While this tool is currently limited to research use, a global methylome database combined with machine learning may improve our current classification of PitNETs. A better understanding of DNA methylation at the genetic level will help to guide the development of new medical strategies to treat PitNETs. With one recent study demonstrating reliable detection of PitNET DNA methylation signatures in the serum and plasma, the future of pituitary epigenomics may see a combination of noninvasive testing and machine learning applications develop unprecedented diagnostic and prognostic models for pituitary tumors.^25^

## Conclusion

While PitNETs do not exhibit many genetic alterations classically associated with other types of neoplasms, epigenetics appears to play a large role in tumorigenesis. Global DNA methylation patterns as well as methylation status of specific genes and DMPs correlate with transcriptome-based PitNET subtype classification. DNA methylation analysis is an important tool in understanding PitNET tumorigenesis, prognostics, and behavior as well as developing targeted therapies.
